# Effects of atrial fibrillation on motor outcome in patients with cerebral infarction

**DOI:** 10.1097/MD.0000000000029549

**Published:** 2022-07-15

**Authors:** Sung Ho Jang, Kyu Hwan Choi

**Affiliations:** a Department of Physical Medicine and Rehabilitation, Yeungnam University Medical Center, Daegu, Republic of Korea.

**Keywords:** atrial fibrillation, cerebral infarction, corticospinal tract, diffusion tensor imaging, motor function

## Abstract

**Background:**

Atrial fibrillation (AF) has been a leading cause of cerebral infarction, but the association with motor outcome after cerebral infarction remains unreported. In this study, we attempted to identify whether AF affects motor outcomes after cerebral infarction.

**Methods:**

Seventy-six patients with a first-incidence cerebral infarction and who completed 6 months of rehabilitation were recruited to this retrospective study. The patients were divided into two groups based on the presence of AF (AF and non-AF groups). The upper extremity motricity index, lower extremity motricity index (LMI), modified Brunnstrom classification, and functional ambulation category (FAC) were evaluated, and those results were obtained within the first day and after 6 months of onset. Clinical factors that could affect motor outcome after cerebral infarction were also obtained.

**Results:**

Compared with the non-AF group, the AF group had an upper extremity motricity index (47.15 ± 20.30 vs 58.66 ± 19.19; *P* = .032), LMI (53.42 ± 12.27 vs 65.58 ± 13.86; *P* = .001), and FAC scores (2.39 ± 0.93 vs 3.35 ± 0.93; *P* < .001) at 6 months after onset. Moreover, the AF group showed a lower FAC score gain than the non-AF group at 6 months after onset (2.33 ± 0.95 vs 3.28 ± 0.94; *P* < .001). Multivariate linear regression analyses showed that presence of AF had negative correlation with LMI gain (β = –0.197; *P* = .010) and FAC gain (β = –0.254; *P* = .011).

**Conclusion:**

We observed that AF had a negative effect on the motor outcome of the affected leg and the recovery of gait function in patients with cerebral infarction.

## 1. Introduction

Cerebral infarction can undermine a patient’s quality of life and is a burden on public health. Approximately 700,000 cases of cerebral infarction occur annually in the United States alone.^[1]^ Cerebral infarction manifests various neurological sequelae, such as motor weakness, spasticity, dysphagia, and cognitive dysfunction.^[2–5]^ Motor weakness after cerebral infarction is closely related to functional decline and deterioration of physical performance and activities of daily living (ADLs).^[2–5]^ Therefore, it is important to identify prognostic factors related to motor outcome after cerebral infarction for predicting postinfarction functional outcome, estimating the duration of rehabilitative therapy, and establishing a rehabilitation strategy.

Many previous studies have documented prognostic factors related to motor outcome in patients with cerebral infarction. Age, location of lesion, leukoaraiosis, intervention procedure during the acute stage, and rehabilitation program have been known to be the prognostic factors for motor outcome following cerebral infarction by previous studies.^[6–11]^ In addition, the state of the corticospinal tract (CST), the most important neural tract related to motor function, is known to be definitively related to motor outcome after cerebral infarction.^[12–15]^ Clinical comorbidities, such as diabetes mellitus, genetic factors, and infection, also affect motor outcome following cerebral infarction.^[11,16]^

Atrial fibrillation (AF) is the most common cardiac arrhythmia in adults and is the leading cause of cerebral infarction, with approximately 24% of patients with cerebral infarction having AF.^[1,17]^ Although there is a high prevalence of AF in patients with cerebral infarction, there are a limited number of previous studies investigating functional outcomes after cerebral infarction.^[18–21]^ These studies have demonstrated that AF could decrease functional outcome after cerebral infarction. However, these previously reported studies investigated only ADLs, using as tools for evaluating functional independence of patients, the functional independence measure (FIM), and modified Barthel index (MBI) in order to assess functional outcome rather than assessing motor outcome directly, and they did not assess important clinical factors related to motor outcome, such as age, location of lesion, clinical comorbidities, and CST state. Therefore, because of the high prevalence of AF in patients with cerebral infarction, it is a priority to determine whether AF affects motor outcome in patients with cerebral infarction.

In this study, we attempted to identify whether AF affects motor outcomes in patients with cerebral infarction. Furthermore, we aimed to evaluate the effect of factors associated with motor outcome to determine the degree to which AF affects motor outcome after cerebral infarction compared to that of other related factors.

## 2. Methods

### 2.1. Participants

A total of consecutive 76 patients (43 men; average age, 62.61 ± 9.89 years; range, 37–79 years) were recruited according to the following inclusion criteria: first-ever stroke; unilateral cerebral infarction with a supratentorial white matter lesion diagnosed by magnetic resonance imaging; definite hemiparesis: severe motor weakness of the affected extremities resulting in the inability to move the affected extremities without gravity and complete motor weakness of the affected hand (finger flexor and extensor) within the first day of cerebral infarction onset; diffusion tensor imaging (DTI) scanning performed to identify disruption of CST integrity within 7 ~ 30 days from onset; absence of severe cognitive dysfunction (Mini-Mental State Examination score <20); no serious medical condition (e.g., pneumonia or sepsis) or neurological or musculoskeletal problems from onset to final evaluation; no history of other cardiac problems except AF (e.g., ischemic heart disease, valvular heart disease, or heart failure with reduced ejection fraction under 40%); and no surgical intervention performed for cerebral infarction (e.g., catheterization or craniectomy). All recruited patients who met the inclusion criteria underwent 6 months of rehabilitation. Rehabilitation was performed for 150 minutes 6 d/wk (Saturday, 75 min/d) after onset, and the rehabilitation program consisted of physical therapy, occupational therapy, and neuromuscular electrical stimulation of the affected extremity muscles.

Clinical information on factors that could affect motor outcome after cerebral infarction was obtained at admission (within the first day of onset). The obtained clinical information included age, hypertension, diabetes mellitus, dyslipidemia, obesity, and AF. The recruited patients were divided into two groups based on the presence of AF (AF and non-AF groups). Patients with paroxysmal, persistent, or permanent AF were included in the AF group.^[22,23]^ Detailed demographic and clinical characteristics of the patients are listed in Table 1. Of the 76 recruited patients, 18 (23.7%) patients had AF. The age, sex, hypertension, diabetes mellitus, dyslipidemia, and obesity characteristics of the two groups were not statistically different between the AF and non-AF groups (*P* ≥ .05).

**Table 1:** Demographic and clinical characteristic of cerebral infarction patients by atrial fibrillation.

Variable	Total patients	AF group	Non-AF group	*P*-value
N (%)	76	18 (23.7)	58 (76.3)	
Age (yr)*	62.61 (9.89)	65.50 (10.17)	61.71 (9.72)	.157
Male, n (%)	43 (56.6)	11 (61.1)	32 (55.2)	.657
Hypertension, n (%)	48 (63.2)	13 (72.2)	35 (60.3)	.361
Diabetes mellitus, n (%)	21 (27.6)	8 (44.4)	13 (22.4)	.068
Dyslipidemia, n (%)	26 (34.2)	3 (16.7)	23 (39.7)	.073
Obesity, n (%)	27 (35.5)	7 (38.9)	20 (34.5)	.733
Lesion side, right, (%)	35 (46.1)	7 (38.9)	28 (48.3%)	.485
Duration to DTI (d) †	14.16 (7.39)	17.90 (9.25)	12.98 (6.35)	.051
Preserved CST, n (%)	61 (80.3)	12 (66.7)	49 (84.5)	.097

AF = atrial fibrillation; CST = corticospinal tract; DTI = diffusion tensor imaging.

* Values are presented as mean (standard deviation) values.

### 2.2. Clinical evaluation

Assessments of motor function of the affected extremities were performed twice (within the first day of onset and after completing 6 months of rehabilitation). Motor function of the affected extremities was evaluated using the motricity index (MI; maximum score, 100).^[24]^ The MI for the extremities was described as upper extremity MI (UMI) for the affected arm or lower extremity MI (LMI) for the affected leg.^[24]^ The motor function of the affected hand was evaluated by determining the modified Brunnstrom classification (MBC) (score range, 1–6).^[25]^ The ability to walk with or without assistance was evaluated by determining the functional ambulation category (FAC; score range, 0–5).^[26]^ Higher MI, MBC, and FAC scores imply a better motor outcome.

### 2.3. DTI scan

The DTI scanning was conducted at 14.16 ± 7.39 days after the onset of cerebral infarction. Scans were obtained using a sensitivity-encoding head coil on a Philips Gyroscan Intera 1.5T MRI (Hoffman-LaRoche, Ltd, Best, The Netherlands) with single-shot echo-planar imaging and navigator echo. Sixty contiguous slices were acquired. The DTI scan parameters were set as follows: matrix = 128 × 128 matrix; field of view = 221 mm × 221 mm; echo time = 76 ms; repetition time = 10,726 ms; parallel image reduction factor = 2; echo-planar image factor = 67; number of excitations = 1; and a 2.3-mm slice thickness. All setting parameters were used for each of the 32 noncollinear diffusion-sensitizing gradients. Head motion effects and image distortions owing to eddy currents were corrected by applying multiscale affine 2-dimensional (2D) registration.

The preservation of the integrity of the CST was assessed by performing fiber assignment using the 3-dimensional fiber reconstruction algorithm within the Philips PRIDE software (Philips Medical Systems, Ltd, Best, The Netherlands), which allows continuous tracking. A seed region of interest (ROI) was drawn in the CST portion of the anterior mid-pons on a 2D fractional anisotropy (FA) color map. Another ROI was also drawn in the CST portion of the anterior low-pons on a 2D FA color map. The CST tracking was performed using an FA threshold >0.2 and a direction threshold of 60°.^[27]^ The tract fibers passing through both ROIs were designated tracts of interest. Preservation of CST integrity meant that the tract from the motor cortex of the affected hemisphere was preserved and the CST passed around the cerebral infarction lesion. The percentages of preserved CST integrity were not significantly different between the AF and non-AF groups (*P* ≥ .05).

### 2.4. Statistical analysis

The collected data were statistically analyzed using the Statistical Package for the Social Sciences software ver. 23.0 (IBM Corporation, Released 2019, Armonk, NY). Comparisons of demographic, clinical factors, and motor outcomes between the AF and non-AF groups were tested using the Mann–Whitney *U* test for continuous variables and the Chi-square test for categorical variables. Multivariate linear regression analysis was used to determine the association between motor gain in the 6 months after cerebral infarction onset and factors that could affect motor outcome and initial motor function. The variables included in the regression model were age, CST preservation state, presence of AF, hypertension, diabetes mellitus, dyslipidemia, obesity, and initial motor function after cerebral infarction. Herein, we present the regression coefficients and *P*-values for each variable. We presented β, *R*^2^, adjusted *R*^2^, and *P*-values for each regression model to evaluate the coefficient of determination and statistical significance. Considering the number of variables in the regression model, we presented adjusted *R*^2^ to evaluate how the model explains the variability of dependent variable (motor gain). In our study, the statistical significance level was set at *P* < .05.

### 2.5. Ethics statement

This retrospective study protocol was reviewed, and an exemption of informed consent was approved by the institutional review board of a university hospital.

## 3. Results

There were no significant differences in initial motor function in terms of UMI, LMI, MBC, and FAC scores between the AF and non-AF groups, and all patients showed significant improvement in all motor function scores (UMI, LMI, MBC, and FAC) at 6 months after onset (Table 2). However, comparisons of motor outcomes of the AF and non-AF groups showed that the AF group had lower UMI (47.15 ± 20.30 vs 58.66 ± 19.19; *P* = .032), LMI (53.42 ± 12.27 vs 65.58 ± 13.86; *P* = .001), and FAC (2.39 ± 0.93 vs 3.35 ± 0.93; *P* < .001) scores than the non-AF group at 6 months after onset. Differences between the two groups in motor gain during the 6 months after infarction were observed only in the FAC score, with a lower FAC gain in the AF group than that in the non-AF group (2.33 ± 0.95 vs 3.28 ± 0.94; *P* < .001).

**Table 2:** Comparison of motor outcomes of cerebral infarction patients by atrial fibrillation.

Variable*	AF group	Non-AF group	*P*-value
Initial UMI	2.17 (9.19)	6.81 (13.92)	.109
Initial LMI	4.92 (14.35)	12.02 (17.86)	.094
Initial MBC	1.06 (0.24)	1.05 (0.22)	.950
Initial FAC	0.06 (0.24)	0.07 (0.26)	.844
UMI at 6 mo after onset	47.15 (20.30)	58.66 (19.19)	.032
LMI at 6 mo after onset	53.42 (12.27)	65.58 (13.86)	.001
MBC at 6 mo after onset	2.89 (1.68)	3.59 (1.68)	.123
FAC at 6 mo after onset	2.39 (0.93)	3.35 (0.93)	<.001
UMI gain during 6 mo	44.98 (21.03)	51.84 (20.98)	.230
LMI gain during 6 mo	48.05 (15.20)	53.56 (20.55)	.266
MBC gain during 6 mo	1.83 (1.65)	2.54 (1.68)	.120
FAC gain during 6 mo	2.33 (0.95)	3.28 (0.94)	<.001

AF = atrial fibrillation; FAC = functional ambulation categories; LMI = lower extremity motricity index; MBC = modified Brunnstrom classification; UMI = upper extremity motricity index.

* Values are presented as mean (standard deviation) values.

The results of the multivariate linear regression analysis of motor gain at 6 months after infarction are presented in Table 3. The analyzed motor gain factors explained 36.6% of the UMI gain, 66.1% of the LMI gain, 23.8% of the MBC gain, and 41.6% of the FAC gain at 6 months after onset. In addition, AF was significantly correlated with decreased LMI gain (β = –0.197; *P* = .010) and FAC gain (β = –0.254; *P* = .011). However, AF was not significantly correlated with UMI (β = –0.113; *P* = .267) or MBC gain (β = –0.048; *P* = .669). The preservation of CST integrity had the highest coefficient of correlation with the gains in all four motor outcome scales (UMI gain, β = 0.447; LMI gain, β = 0.321; MBC gain, β = 0.505; and FAC gain, β = 0.418) (*P* < .001). Moreover, age was correlated with a decrease in LMI gain (β = –0.222; *P* = .008) and FAC gain (β = –0.364; *P* = .001). Initial motor functions had negative correlation coefficients with all motor gain indicators at 6 months (initial UMI and 6-month UMI gain, initial LMI and 6-month LMI gain, and initial FAC and 6-month FAC gain).

**Table 3:** Results of multivariate linear regression analysis to evaluate the association between clinical factors and motor gains at 6 mo after cerebral infarction.

Variable	UMI gain		LMI gain		MBC gain		FAC gain	
	β	*P*-value	β	*P*-value	β	*P*-value	β	*P*-value
Atrial fibrillation	–0.113	.267	–0.197	.010	–0.048	.669	–0.254	.011
CST preservation	0.447	<.001	0.321	<.001	0.505	<.001	0.418	<.001
Age	–0.193	.087	–0.222	.008	–0.069	.574	–0.364	.001
Hypertension	0.073	.484	0.123	.110	0.064	.578	0.007	.941
Diabetes mellitus	–0.094	.367	0.006	.938	–0.043	.704	0.043	.670
Dyslipidemia	–0.010	.925	–0.091	.252	0.071	.549	–0.014	.895
Obesity	0.164	.104	0.118	.110	0.108	.324	0.021	.829
Initial UMI	–0.579	.001	–0.091	.447	0.110	.541	–0.061	.700
Initial LMI	–0.025	.865	–0.746	<.001	–0.010	.950	0.204	.143
Initial MBC	0.131	.309	0.049	.602	–0.095	.501	0.133	.282
Initial FAC	–0.035	.764	–0.006	.945	–0.151	.233	–0.337	.003
*P*-value	<.001		<.001		.002		<.001	
*R* ^2^	0.459		0.711		0.349		0.502	
Adjusted *R*^2^	0.366		0.661		0.238		0.416	

CST = corticospinal tract; FAC = functional ambulation categories; LMI = lower extremity motricity index; MBC = modified Brunnstrom classification; UMI = upper extremity motricity index.

## 4. Discussion

In this study, we investigated whether AF affects motor outcomes in patients with cerebral infarction. Our results are summarized as follows: the AF group had lower UMI, LMI, and FAC scores than those of the non-AF group at 6 months after cerebral infarction onset; the AF group showed a lower FAC score gain than that in the non-AF group at 6 months after onset; multivariate linear regression analysis revealed that the presence of AF was a significant independent factor associated with decreased LMI and FAC gains at 6 months after onset, whereas AF was not correlated with UMI or MBC gains at 6 months after onset; older age was also an independent factor for a poor motor outcome and was associated with decreased LMI and FAC gains in our multivariate linear regression analysis; and among the related clinical factors, preservation of CST integrity was the most critical predictive factor for all measured motor gains (UMI, LMI, MBC, and FAC) during the 6 months after onset. Our results demonstrate that AF negatively affects motor outcome in patients with cerebral infarction, and, in particular, the AF-related decrease in motor outcome was most prominent in the affected leg.

There were no significant differences among the baseline demographic and clinical characteristics between the two groups except for the presence of AF (Table 1). However, the AF group had lower average UML, LMI, and FAC scores than those of the non-AF group at 6 months after onset. In addition, we estimated motor gain, which is the difference in motor function between infarction onset and 6 months after onset, to obtain an accurate comparison of postinfarction motor outcomes between the two groups. Compared with the non-AF group, the AF group had a lower gain in FAC score after completing 6 months of rehabilitation. Our result indicates that AF appears to affect negatively gait recovery, a parameter that is closely related to LMI.

Multiple linear regression analysis was used to examine causal relationships between AF presence and motor gain at 6 months after onset, and AF presence was shown to have a negative correlation coefficient in the association with LMI and FAC gains (Table 3). However, AF was not a significant predictor of UMI or MBC gains at 6 months after onset. Our results suggest that patients with AF have decreased motor gain for the affected leg and decreased walking ability gain during the infarction recovery phase compared with that in patients without AF after cerebral infarction. Therefore, our study results indicate that AF can be a risk factor for deteriorated walking ability after cerebral infarction.

There are many reported risk factors that have been related to a poor motor outcome in patients with cerebral infarction, including age, diabetes mellitus, and CST state.^[11–16]^ Therefore, we performed a multivariate linear regression analysis to determine the extent to which factors that affect motor outcome have an effect on motor outcome after infarction by controlling covariates and examining the coefficient of determination. The CST is the most important neural tract related to motor function, and disruption of CST integrity is known to be related to poor motor outcomes in cerebral infarction patients.^[12–15]^ The preservation of CST integrity had the highest correlation coefficient with the increases in motor gain (UMI, LMI, MBC, and FAC scores) in our regression analysis (Table 3), and those results were consistent with those presented in previous studies.^[12–15]^ Patient age was negatively correlated with LMI and FAC gains at 6 months after onset, which is in accordance with previous studies showing that age hinder motor recovery in cerebral infarction patients.^[10,11]^

The mechanisms by which AF hinders motor outcome after cerebral infarction have not been fully determined. In the following, we present several hypotheses for the mechanisms by which AF undermines motor outcome after cerebral infarction. Hypothesis 1: AF could disturb recovery of the neural tracts related to motor function, resulting in a poor motor outcome after cerebral infarction. In the recovery of motor function after cerebral infarction, neural plasticity and reorganization of injured neural tract by cerebral infarction are important.^[28,29]^ AF induces adverse hemodynamic effects on whole-brain perfusion due to beat-to-beat variation.^[30,31]^ Moreover, Stefansdottir et al^[32]^ reported that AF is associated with reduced brain volume. These studies document that AF can induce hemodynamic instability, brain hypoperfusion, and brain volume loss, indicating that neural tract recovery may be disturbed by AF. Hypothesis 2: AF leads to diminished atrial contraction, atrioventricular synchrony, and decreased systolic function of the heart, resulting in reduced cardiac output.^[33]^ Although we excluded participants with significant cardiac problems (e.g., ischemic heart disease or heart failure with reduced ejection fraction under 40%) in this retrospective study, participants with AF might have reduced cardiac output than participants without AF inducing decreased peripheral blood perfusion. Therefore, reduced peripheral blood perfusion provoked by AF-related reduced cardiac output and medication prescribed for AF (e.g., beta-blocker) might induce frailty and muscle fatigue resulting in poor physical performance.^[33,34]^ Hypothesis 3: The prevalence of AF increases with age and is a documented risk factor for poor motor outcomes in cerebral infarction patients.^[10,11]^ Because AF prevalence increases with age, age may be associated with a poor motor outcome in cerebral infarction patients with AF than in patients without AF. Although there was no statistically significant difference between the ages of the AF and non-AF groups, the mean age of the AF group was higher than that of the non-AF group. Therefore, the difference in mean age between the two groups may contribute to the decrease in motor outcome in cerebral infarct patients with AF.

Several studies have reported that AF diminishes physical performance and functional outcomes in the general population, but not in populations of postcerebral infarction patients.^[33,35]^ Despite the high prevalence of AF in patients with cerebral infarction, only a few studies have reported on the effect of AF on functional outcomes after cerebral infarction.^[18–21]^ Karataş et al^[18]^ and Giaquinto et al^[19]^ compared functional outcomes of poststroke patients according to AF status by assessing FIM scores or using an Adapted Patient Evaluation Conference System to demonstrate that poststroke patients with AF had poorer functional independence outcomes than those of patients without AF. Mizrahi et al^[20]^ and Kim et al^[21]^ compared functional outcomes according to the presence of AF by assessing FIM, MBI, and PULSES profiles in patients with cerebral infarction and concluded that postcerebral infarction patients with AF have poorer functional outcomes than those in patients without AF. However, these previous studies have limitations as they assessed only ADLs, such as ambulating, feeding, dressing, communicating, and personal hygiene, by examining FIM Adapted Patient Evaluation Conference System, MBI, and PULSES profiles, which are instruments used to measure the independent functioning of patients rather than measuring motor function specifically. Hence, these past studies could not present a detailed assessment of motor outcome after cerebral infarction. To the best of our knowledge, the present study is the first to demonstrate the effects of AF on motor outcome after cerebral infarction while considering the state of integrity of the CST.

However, our study has some limitations. First, our study recruited a relatively small number of patients. Although there were differences in the proportion of the demographic and clinical data including age, hypertension, and diabetes mellitus between groups (AF vs Non-AF group), there were no statistically significant differences (Table 1). We think that the small sample size of our study might affect these statistical results. Second, this study did not include the motor function-related neural tracts other than the CST. Finally, because our study was designed as a retrospective study, limited information on the patients was available. To overcome these limitations, well-controlled prospective involving a larger number of subjects are needed. Furthermore, because the mechanisms how AF affects motor outcome after cerebral infarction have not been clearly elucidated, further studies including instruments to measure cardiovascular functional class and physiologic indicators to examine the several hypotheses should be invited.

## 5. Conclusion

We observed that the presence of AF had a negative effect on motor outcome of the affected leg and recovery of gait function in patients with cerebral infarction. Therefore, clinicians should be aware that AF can be a risk factor for poor motor outcome in the affected leg and walking ability in cerebral infarction patients. Consequently, rehabilitation programs for motor facilitation for the affected leg and gait training are needed during recovery after cerebral infarction in patients with AF.

## Author contributions

Conceptualization: Sung Ho Jang.

Data curation: Kyu Hwan Choi, Sung Ho Jang.

Funding acquisition: Sung Ho Jang.

Methodology: Kyu Hwan Choi.

Project administration: Sung Ho Jang.

Supervision: Sung Ho Jang.

Visualization: Sung Ho Jang.

Writing – original draft: Kyu Hwan Choi.

Writing – review & editing: Sung Ho Jang.
